# Demographic and prognostic landscape of upper urinary tract urothelial carcinoma: an analysis of a national cancer database

**DOI:** 10.3389/fruro.2025.1647133

**Published:** 2026-01-16

**Authors:** Beau Hsia, Mark Moroz, Ethan Sipes, Amber Chang, Rania Jundi, Susan Rafie, Peter Silberstein, Abubakar Tauseef

**Affiliations:** 1 Creighton University School of Medicine, Phoenix, AZ, United States; 2 Grand Canyon University, Phoenix, AZ, United States; 3 University of South Florida Morsani College of Medicine, Tampa, FL, United States; 4 Arizona State University, Tempe, AZ, United States; 5 Midwestern University Arizona College of Osteopathic Medicine, Glendale, CA, United States; 6 CHI Health Creighton University Medical Center, Omaha, NE, United States

**Keywords:** NCDB, primary anatomical site, prognostic variables, urogenital system, urothelial carcinoma (UC)

## Abstract

**Purpose:**

Upper urinary tract urothelial carcinoma (UTUC) is an uncommon malignancy of the urogenital tract with a wide range of clinical outcomes. While prognostic factors for bladder-based UC are established, less is known about tumors in other locations and the impact of socioeconomic disparities. This study uses a large national database to identify key demographic, clinical, and socioeconomic predictors of overall survival in UC patients, focusing on the primary tumor site.

**Methods:**

We conducted a retrospective analysis of 12,300 patients diagnosed with UC of the kidney and renal pelvis between 2004 and 2020 from the National Cancer Database (NCDB). Multivariable Cox proportional hazards regression was used to analyze the association between overall survival and factors including patient demographics, tumor characteristics, primary tumor site, and socioeconomic status.

**Results:**

The cohort was predominantly male (59%) and White (91%), with a mean age of 71 at diagnosis. Multivariable analysis identified several factors significantly associated with survival. Renal pelvis tumors, the most common primary site (84.9%), were associated with significantly improved survival (HR = 0.84; 95% CI: 0.8–0.9; p<0.001). Compared to males, females exhibited a 15% lower hazard of death (HR = 0.85; 95% CI: 0.81-0.90; p<0.001). Factors associated with worse survival included a higher Charlson-Deyo comorbidity score (HR = 1.51; 95% CI: 1.39-1.65; p<0.001) and advanced NCDB tumor stage. Socioeconomically, patients with higher income (HR = 0.82; 95% CI: 0.75-0.90; p<0.001) and private insurance or Medicare (HR = 0.70; 95% CI: 0.57-0.87; p<0.001) had improved survival. Adjuvant chemotherapy was associated with a lower hazard of death (HR = 0.84; 95% CI: 0.75-0.95; p=0.007), whereas primary radiation therapy was associated with a higher hazard of death (HR = 1.69; 95% CI: 1.54-1.86; p<0.001).

**Conclusion:**

This large-scale analysis identifies the primary tumor site as a key prognostic factor in UC, with renal pelvis tumors demonstrating more favorable survival. The study also confirms the significant influence of comorbidity and tumor stage while uniquely highlighting that socioeconomic factors, such as income and insurance, are powerful predictors of outcome. These findings underscore the need for optimized, site-specific treatment strategies and concerted efforts to address healthcare inequities in the management of upper tract UC.

## 1 Introduction

Upper urinary tract urothelial carcinoma (UTUC), also previously known as papillary transitional cell carcinoma (PTCC), is a neoplasm of the urothelium characterized by papillary structures exhibiting varying degrees of cytoarchitectural atypia (1). It is distinguished from other urothelial neoplasms by its papillary configuration and is further classified into low-grade and high-grade variants. The papillary architecture ranges from elongated and organized to complex, exhibiting solid or fused formations.

UC constitutes less than 4% of urothelial neoplasms and is characterized by papilla, or finger-like projections, originating from the inner lining of the bladder (1). Recurrence rates for UC are high, ranging from 30.4% to 71%, while progression rates to more aggressive disease occurs in 2.4% and 9% of cases (1–5). Although most frequently arising in the bladder, UC has also been identified in other regions of the urinary tract, including the renal pelvis and ureter (6).

Standard treatment typically involves transurethral resection of the tumor. Due to high recurrence rates, patients often require long-term surveillance and adjuvant intravesical therapies such as Bacillus Calmette-Guérin (BCG) or mitomycin C (1, 6). Data on long-term survival is limited, but five-year survival rates for bladder-confined UC range from 78% to 94%. Survival outcomes for UC in extravesical locations are less well-documented. Identified risk factors include smoking, chemical carcinogen exposure, female sex, and Black race (1, 7, 8).

Despite these clinical insights, there remains a significant gap in comprehensive, population-based analyses investigating prognostic factors associated with overall survival in UC. This study aims to address that gap by evaluating the relationship between overall survival in UC and a comprehensive set of variables. We analyzed the impact of primary tumor location alongside key socioeconomic, demographic, and prognostic factors. Variables analyzed include Charlson-Deyo (CD) comorbidity index, primary payer (insurance), sex, age, race, median income, education level, American Joint Committee on Cancer (AJCC) stage, primary radiation therapy, primary chemotherapy, primary immunotherapy, and adjuvant chemotherapy.

## 2 Materials and methods

This retrospective study analyzed patients diagnosed with UC from 2004 to 2020 utilizing data from the National Cancer Database (NCDB). The NCDB is a nationwide clinical oncology registry jointly sponsored by the American College of Surgeons and the American Cancer Society, capturing approximately 70% of newly diagnosed cancers in the United States. It includes information on patient characteristics, tumor staging, histology, initial treatment, disease recurrence, and survival from over 1,500 Commission on Cancer-accredited facilities. De-identified patient data were accessed by the authors through the Participant User Data Files program. Patients with UC were identified using the ICD-O-3 histology code 8130, which classifies tumors based on morphological and histological features, and Primary Site codes C64 (Kidney, NOS) and C65 (Renal Pelvis). Exclusion criteria were applied sequentially: patients with primary sites outside the kidney/renal pelvis (e.g., bladder) were excluded to focus on upper tract disease; patients with concurrent tumors or missing clinical/demographic data were subsequently excluded.

### 2.1 Covariates

Patient characteristics included age, sex, race, ethnicity, education level, median household income, and insurance status. Clinical variables included tumor size, NCDB analytical stage, Charlson-Deyo (CD) comorbidity score, primary tumor site, primary radiation therapy, primary/adjuvant chemotherapy, and distance traveled for healthcare in miles. Race was categorized as White, African American, or Other, with the latter including American Indian/Alaska Native, Asian (e.g., Chinese, Japanese, Filipino, etc.), and Pacific Islander groups. Ethnicity was classified as Hispanic or non-Hispanic, per NCDB standards. Income was represented by median household income (2016-2020) based on the patient’s residential zip code at diagnosis. Education level was defined as the percentage of residents in the patient’s zip code (2020 data) who did not complete high school. Tumor stage (I-IV) was determined via NCDB analytical stage. Insurance was categorized as uninsured, private, Medicare, Medicaid, or other government insurance. Primary anatomic site was categorized into kidney, renal pelvis, and other tissues. Distance traveled for healthcare was calculated as the mileage between the patient’s residence and the reporting hospital. The Charlson-Deyo (CD) comorbidity score stratified patients into groups of 0, 1, 2, and ≥3. Primary chemotherapy and radiation were defined as systemic or regional therapy administered as part of the first course of treatment. Adjuvant chemotherapy was defined as systemic therapy administered following definitive surgical resection.

### 2.2 Outcomes and analysis

The primary outcome was overall survival (OS), defined as the time from diagnosis to death, with censoring at the last contact date. Prognostic factors were identified using multivariable Cox proportional hazards regression. Kaplan-Meier curves and life tables were used to estimate OS at 2,5, and 10 years. The proportional hazards assumption was assessed using log-negative-log survival curves and tests for statistical interaction with time. A robust sandwich covariance matrix was used to account for patient clustering within facilities.

### 2.3 Statistical methods

Cox proportional hazards regression was chosen for its ability to handle censored data and evaluate multiple prognostic factors simultaneously. Kaplan-Meier curves visualized unadjusted survival differences, while life tables provided standardized survival estimates at clinically relevant timepoints (2, 5, and 10 years) as mentioned above. These methods align with NCDB and American Joint Committee on Cancer (AJCC) recommendations for cancer registry analyses. Descriptive statistics, unadjusted survival analysis, and multivariable analysis were performed using IBM SPSS version 27 (IBM Corp., Armonk, NY). Patients with missing clinical or demographic data were excluded. A Bonferroni correction adjusted the p-value threshold for multiple comparisons to p<0.0083 (0.05/6), maintaining a family-wise error rate of α=0.05. This conservative approach was preferred over false discovery rate control due to the exploratory nature of socioeconomic factor analyses. Multicollinearity among socioeconomic variables (income, education, insurance status) was assessed using variance inflation factors (VIF) from linear regression models, with all VIFs <2.0, well below the threshold of 5.0, indicating no significant collinearity.

## 3 Results

A study group of 12,300 patients was analyzed. Descriptive statistics are displayed in **Tables 1**–**3**. The population was predominantly male (59%) and White (91%). The mean age at diagnosis was 71. Most patients (67%) were covered by Medicare insurance and a large portion fell within the highest income bracket (≥$75,000, 38%). The Charlson-Deyo score indicated a generally healthy population, with 66.5% of patients having a score of zero. NCDB stage I was the most common diagnosis (43.1%), and the renal pelvis was the most frequent tumor site (84.9%). Surgical intervention was performed in 88% of patients, followed by primary chemotherapy (18.7%), and adjuvant chemotherapy (10.7%). Residual tumors were undetectable in 88% of cases.

**Table 1:** Clinical and demographic characteristics of 12,300 patients with upper urinary tract urothelial carcinoma.

Variable	N=12,300	% of total
Sex		
Male	7310	59.4
Female	4990	40.6
Race		
White	11169	90.8
Black	667	5.4
Other	464	3.8
Age (years)		
Mean ± Standard deviation	71.15 ± 11.20	N/A
Median (interquartile range)	72.0 (64.0-80.0)	N/A
Zip code-level median household income (2016-2020, $)		
< $46,277	1807	14.7
$46,277-$57,856	2704	22
$57,857-$74,062	3073	25
≥ $74,063	4716	38.3
Zip code-level education (% without high-school degree, 2020)		
≥ 15.3%	2233	18.2
9.1%-15.2%	3460	28.1
5%-9%	3742	30.4
< 5%	2865	23.3
Insurance status		
Uninsured	203	1.7
Private	3281	26.7
Medicaid	426	3.5
Medicare	8273	67.3
Other government	117	1
Distance traveled for health care (miles)		
Mean ± Standard deviation	25.022 ± 64.3394	N/A
Median (interquartile range)	10.1 (0.0-20.3)	N/A
Charlson-Deyo comorbidity score		
0	8178	66.5
1	2673	21.7
2	544	7.4
≥ 3	95	4.4
Tumor size (mm)		
Mean ± Standard deviation	43.4887 ± 33.38245	N/A
Median (interquartile range)	38.0 (23.5-52.5)	N/A
NCDB analytical stage		
I	3766	53.2
II	634	9
III	1558	22
IV	1121	15.8
Primary Anatomic site		
Kidney, NOS	1791	14.6
Renal Pelvis	10446	84.9
Other	63	0.5
Primary therapy		
Received primary radiation therapy	610	5
Received primary chemotherapy	2296	18.7
Received primary immunotherapy	214	1.7
Adjuvant therapy	N/A	N/A
Received adjuvant chemotherapy	1321	10.7
Surgical Margins		
No residual tumor	10262	83.4
Residual tumor, NOS	260	2.1
Microscopic residual tumor	329	2.7
Macroscopic residual tumor	58	0.5

NCDB, National Cancer Database; N/A, Not applicable; NOS, Not otherwise specified.

**Table 2:** Median two, five, and ten-year survival estimates of 12,300 patients with upper urinary tract urothelial carcinoma.

Variable	2-year (%)	5-year (%)	10-year (%)
Sex			
Male	72.6	52.2	33.3
Female	74.2	53.8	36.4
Race			
White	73.2	52.6	34.3
Black	72.8	53.7	36.1
Other	75.2	57.7	40.7
Zip code-level median household income (2016-2020, $)			
< $46,277	71.5	50.7	30.4
$46,277-$57,856	70.5	50.5	31.8
$57,857-$74,062	72.9	52.8	33.8
≥ $74,063	75.7	55	38.4
Zip code-level education (% without high-school degree, 2020)			
≥ 15.3%	71.7	52.3	33.9
9.1%-15.2%	71.8	50.8	31.1
5%-9%	74.7	53.7	35.6
< 5%	74.3	54.6	38
Age (years)			
0-25	50	50	N/A
26-50	84.40	71.9	61.8
51-75	79.2	61.4	45.6
76-100	63.4	38.4	15.6
NCDB analytical stage			
I	85.3	65.6	44.1
II	81.3	56.9	35.1
III	71.4	48.9	31.3
IV	33.7	18.4	11.3

NCDB, National Cancer Database.

**Table 3:** Multivariable cox regression model of 12,300 patients with upper urinary tract urothelial carcinoma.

Variable	HR (95% confidence interval)	P values
Age (5 years)	1.25 (1.24 - 1.27)	<0.001
Males (Ref.) vs. females	0.85 (0.81 - 0.90)	<0.001
Race and ethnicity		
White (Ref.) vs. Black	1.07 (0.95 - 1.19)	0.26
White (Ref.) vs. Other	0.85 (0.74 - 0.98)	0.023
Black (Ref.) vs. Other	0.80 (0.67 - 0.95)	0.011
Charlson-Deyo comorbidity score		
0 (Ref.) vs. 1	1.18 (1.11 - 1.25)	<0.001
0 (Ref.) vs. 2	1.51 (1.39 - 1.65)	<0.001
0 (Ref.) vs. ≥ 3	1.49 (1.33 - 1.68)	<0.001
1 (Ref.) vs. 2	1.28 (1.17 - 1.41)	<0.001
1 (Ref.) vs. ≥ 3	1.27 (1.12 - 1.43)	<0.001
2 (Ref.) vs. ≥ 3	0.99 (0.86 - 1.13)	0.843
Zip code-level median household income (2020 US Dollars)		
< $46,277 (Ref.) vs. $46,227-$57,856	0.95 (0.87 - 1.03)	0.212
< $46,277 (Ref.) vs. $57,857-$74,062	0.91 (0.84 - 1.00)	0.039
< $46,277 (Ref.) vs. ≥ $74,063	0.82 (0.75 - 0.90)	<0.001
$46,227-$57,856 (Ref.) vs. $57,857-$74,062	0.96 (0.90 - 1.03)	0.304
$46,227-$57,856 (Ref.) vs. ≥ $74,063	0.87 (0.81 - 0.93)	<0.001
$57,857-$74,062 (Ref.) vs. ≥ $74,063	0.90 (0.84 - 0.96)	0.003
Zip code-level education (2020, % No high-school diploma)		
≥ 15.3% (Ref.) vs. 9.1%-15.2%	1.02 (0.95 - 1.10)	0.568
≥ 15.3% (Ref.) vs. 5.0%-9.0%	1.01 (0.93 - 1.09)	0.823
≥ 15.3% (Ref.) vs. < 5.0%	0.97 (0.88 - 1.06)	0.497
9.1%-15.2% (Ref.) vs. 5.0%-9.0%	0.99 (0.92 - 1.05)	0.712
9.1%-15.2% (Ref.) vs. < 5.0%	0.95 (0.88 - 1.03)	0.185
5.0%-9.0% (Ref.) vs. < 5.0%	0.96 (0.89 - 1.03)	0.257
Insurance		
None (Ref.) vs. Private	0.70 (0.57 - 0.87)	<.001
None (Ref.) vs. Medicaid	0.83 (0.65 - 1.07)	0.16
None (Ref.) vs. Medicare	0.73 (0.59 - 0.90)	0.003
None (Ref.) vs. Other government	0.69 (0.49 - 0.97)	0.033
Private (Ref.) vs. Medicaid	1.19 (1.01 - 1.40)	0.036
Private (Ref.) vs. Medicare	1.04 (0.97 - 1.11)	0.312
Private (Ref.) vs. Other government	0.98 (0.74 - 1.30)	0.91
Medicaid (Ref.) vs. Medicare	0.87 (0.74 - 1.02)	0.091
Medicaid (Ref.) vs. Other government	0.83 (0.61 - 1.13)	0.237
Medicare (Ref.) vs. Other government	0.95 (0.72 - 1.25)	0.712
Treatment		
No primary radiation therapy (Ref.) vs. primary radiation therapy	1.69 (1.54 - 1.86)	<0.001
No primary chemotherapy (Ref.) vs. primary chemotherapy	0.91 (0.82 - 1.00)	0.057
No primary immunotherapy (Ref.) vs. primary immunotherapy	0.88 (0.71 - 1.09)	0.234
No adjuvant chemotherapy (Ref.) vs. adjuvant chemotherapy	0.84 (0.75 - 0.95)	0.007
NCDB analytical stage		
Stage I (Ref.) vs. Stage II	1.32 (1.21 - 1.44)	<0.001
Stage I (Ref.) vs. Stage III	1.63 (1.53 - 1.73)	<0.001
Stage I (Ref.) vs. Stage IV	5.06 (4.69 - 5.46)	<0.001
Stage II (Ref.) vs. Stage III	1.24 (1.13 - 1.34)	<0.001
Stage II (Ref.) vs. Stage IV	3.84 (3.48 - 4.22)	<0.001
Stage III (Ref.) vs. Stage IV	3.11 (2.89 - 3.35)	<0.001
Primary anatomic site		
Kidney, NOS (Ref.) vs. Renal pelvis	0.84 (0.79 - 0.90)	<0.001
Kidney, NOS (Ref.) vs. Other	0.48 (0.33 - 0.70)	<0.001
Renal pelvis (Ref.) vs. Other	0.57 (0.39 - 0.83)	0.003

NCDB, National Cancer Database; NOS, Not otherwise specified; Ref, Reference group.

The five-year survival rate was 52% for males and 54% for females. Ten-year survival rates were 33% for males and 36% for females, respectively. Females exhibited a lower hazard of death compared to males (Hazard Ratio [HR]=0.85, 95% Confidence Interval [95% CI]: 0.81-0.90, P<0.001). Higher Charlson-Deyo scores were associated with a higher hazard of death compared to a score of zero (e.g., for a score of 2, HR = 1.51, 95% CI: 1.39-1.65, P<0.001). Higher income levels were associated with a lower hazard of death. For example, the highest income bracket (≥$74,000) had a lower hazard of death compared to those in the lowest bracket (<$46,000) (HR = 0.82, 95% CI: 0.75-0.90, P<0.001). Patients with private insurance had a lower hazard of death compared to those who were uninsured (HR = 0.70, 95% CI: 0.57-0.87, P<0.001). The use of adjuvant chemotherapy was associated with a lower hazard of death (HR = 0.84, 95% CI: 0.75-0.95, P = 0.007), while primary radiation therapy was associated with a higher hazard of death (HR = 1.69, CI: 1.54-1.86, P<0.001) (9). Patients with renal pelvis tumors had a lower hazard of death compared to those with a primary site identified as ‘kidney, not otherwise specified’ (HR = 0.84, 95% CI: 0.79-0.90, P<0.001). Conversely, patients with other primary sites had a lower hazard of death compared to those with a primary site in the ‘kidney, NOS’ (HR = 0.48, 95% CI: 0.33-0.70, P<0.001).

No significant association with survival was observed for race, education level, primary chemotherapy treatment, or primary immunotherapy.

## 4 Discussion

This study aimed to provide a comprehensive analysis of prognostic factors in upper tract urothelial carcinoma (UC), with a focus on the significance of primary anatomic site. Our findings corroborate the trend of male predominance in UC (1, 2), although our observed male-to-female ratio of approximately 5:3 is lower than the previously reported range of 3:1 to 8:1 (1). Despite this discrepancy, we identified a statistically significant difference in 5- and 10-year survival rates between sexes, with females exhibiting a lower risk of mortality compared to males. This contrasts with prior research that suggested a higher risk of death for women (8), though this study broadly examined transitional cell carcinoma rather than specifically focusing on UC.

Our observed 5-year survival rate of 52.8% is lower than the 78-94% reported in prior studies (10–12). This difference may be attributed to the fact that earlier research primarily concentrated on bladder-based UC, whereas the majority of patients in our inquiry presented with primary tumors in the renal pelvis. This interpretation is supported by our findings, which demonstrate significantly higher survival rates among patients with renal pelvis involvement.

A substantial portion of existing literature concentrates on UC affecting the bladder (1–4, 6–8, 10, 11). Our study specifically targeted UC arising in the kidney and renal pelvis to address the paucity of data regarding this histology in the upper tract in previous studies (5, 13–15). Consequently, the renal pelvis was the most common primary site in our inquiry (84.9%). The scarcity of research on upper urinary tract UC is often attributable to the aggregation of cancer statistics with other renal malignancies (13), demonstrating the necessity for site-specific analyses. Notably, we found that the renal pelvis as a primary site was associated with a lower hazard of death compared to kidney, NOS tumors—a finding that, to our knowledge, has not been previously reported.

The National Cancer Database (NCDB) staging system classifies disease progression and metastatic spread. Our analysis demonstrates a strong inverse relationship between NCDB stage and survival, consistent with previous reports (8, 10, 11, 16). While one study examining renal pelvis and kidney cancers did not identify stage as a significant predictor of survival (13), this may be due to a limited sample size. To our knowledge, this study is the first to specifically analyze NCDB stages as prognostic indicators in UC. Additionally, our findings confirm that comorbidity, as measured by the Charlson-Deyo score, is a significant predictor of survival, aligning with prior literature (8, 11).

Transurethral resection, followed by intravesical therapy with Bacillus Calmette-Guérin (BCG) or mitomycin C, remains the standard treatment approach (1, 2, 5). This is reflected in our dataset, in which 88% of patients underwent surgical tumor removal. While the efficacy of combined transurethral resection and BCG therapy is supported by existing evidence (2, 5), a smaller proportion of our patient population received primary chemotherapy (18.7%) and adjuvant chemotherapy (10.7%), a finding consistent with other studies (2, 5). A recent meta-analysis of upper urinary tract urothelial carcinoma, including UC, investigated the effectiveness of BCG and mitomycin C, suggesting suboptimal outcomes (17) and proposing that novel drugs and administration techniques may offer promise for future adjuvant therapies (17). In contrast to prior reports, our data indicate that patients who received adjuvant chemotherapy had a lower hazard of death. Notably, primary chemotherapy did not emerge as a significant predictor of survival, further underscoring the potential benefit of adjuvant treatment in this context. Conversely, patients undergoing primary radiation therapy demonstrated a higher hazard of death. This association is likely driven by confounding by indication; in the management of upper tract urothelial carcinoma, radiation is rarely used as a curative intent first-line therapy and is typically reserved for patients with unresectable disease, extensive metastases, or those medically unfit for surgery. Therefore, this group represents a cohort with inherently poorer prognosis.

Socioeconomic factors have been identified as important prognostic indicators in UC and related cancers through cancer registries (11, 18–21). However, to our knowledge, this is the first analysis of socioeconomic factors in UC using NCDB data. Our results show that patients in the highest income bracket had a lower hazard of death compared to those in the lowest income bracket. This contrasts with a Danish study (11), which reported reduced mortality risk in UC patients within the second and third lowest income quintiles. Another broader study of urothelial carcinoma did not find income to be a significant predictor of survival (19). In our analysis, patients with private insurance or Medicare had a significantly lower hazard of death compared to those who were uninsured. There was a suggestion that patients with Medicaid had a higher hazard of death compared to those with private insurance. This finding is supported by a study demonstrating significantly higher disease-specific and all-cause mortality rates in patients with low socioeconomic status (SES) insurance (public insurance) compared to those with high SES insurance (private insurance) (19). Consistent with prior research (19), our study did not find education level to be associated with survival in UC. Research is warranted to explore socioeconomic factors in UC across all primary sites for more comprehensive comparisons.

### 4.1 Limitations

The NCDB’s retrospective design limits access to detailed clinical variables such as specific treatment regimens, their sequencing, and the precise timing of interventions, thereby restricting a granular analysis of optimal therapeutic strategies. Missing data may introduce bias, particularly if the data are not missing at random, and residual confounding from unmeasured factors—such as tumor molecular characteristics or treatment sequence—cannot be ruled out. Furthermore, the NCDB does not report cause-specific mortality data, complicating interpretation of treatment outcomes, especially in older patients with competing risks. Since the NCDB reports only overall survival, attributing deaths directly to UC is not possible. It is crucial to acknowledge that our findings are based on a U.S.-only cohort from Commission on Cancer-accredited facilities, which may introduce selection bias and limit the direct generalizability of these results to international populations or different healthcare settings. However, overall survival remains a meaningful endpoint in large-scale, population-based research. Therefore, future prospective, international studies would be invaluable for validating these findings and exploring the nuances of treatment efficacy. Finally, the use of ‘Kidney, NOS’ as a reference variable presents a limitation due to the ambiguity of the code. While it serves as a baseline for non-specific localization, it lacks the anatomical precision of ‘Renal Pelvis,’ and survival comparisons should be interpreted with this coding constraint in mind.

Despite these limitations, this study has notable strengths. Given the rarity of UC, our moderate sample size offers greater statistical power than many single-institution studies. Our findings are consistent with existing literature, particularly regarding the prognostic significance of stage and primary site, which supports the validity and generalizability of our results within the NCBD’s scope.

## 5 Conclusion

This study identifies primary tumor site as the key prognostic factor in UC, with renal pelvis tumors associated with significantly improved survival. Sex, Charlson-Deyo score, and NCDB stage also significantly influenced outcomes. While confirming previous findings, this study uniquely highlights socioeconomic disparities in UC outcomes. This study has inherent limitations due to its reliance on the NCDB, including a lack of detailed treatment data and cause-specific mortality. Despite these constraints, our analysis provides valuable insights into the prognosis of UC. These findings underscore the need for optimized treatment strategies and for efforts to address healthcare inequities.

## Data Availability

Publicly available datasets were analyzed in this study. This data can be found here: https://www.facs.org/quality-programs/cancer-programs/national-cancer-database/.
